# The structure-preserving spectral graph neural network for dual kinase inhibitors and synergy scoring in gastric cancer

**DOI:** 10.1038/s41746-025-02240-7

**Published:** 2025-12-20

**Authors:** Yang Zhang, Chunhong Yuan, Longgang Wang, Yujia Chen, Yanpeng Xing, Yuanlin Sun

**Affiliations:** 1 Department of Gastrocolorectal Surgery, General Surgery Center, The First Hospital of Jilin University, Changchun, Jilin China; 2 Faculty of Control Systems and Robotics, ITMO University, St. Petersburg, Russia; 3 Department of Gastrointestinal Surgery, Shandong Cancer Hospital and Institute, Shandong First Medical University and Shandong Academy of Medical Sciences, Jinan, Shandong China

**Keywords:** Cancer, Computational biology and bioinformatics, Drug discovery

## Abstract

The therapeutic targeting of kinase signaling pathways represents a pivotal strategy in gastric cancer, yet the rational design of single agents capable of dual-kinase inhibition remains a challenge in precision oncology. Here, we develop the **DuoKinaseNet**, a dual-task spectral graph neural network that integrates global topological information from a heterogeneous biomedical graph to enable structure-preserving prediction of drug–kinase interactions. The core innovation of our model is the Structure-Preserving Spectral Expansion (SPSE) module, which injects global graph topology from a biomedical knowledge graph into the learning process via spectral coordinates and diffusion-distance biased attention. Evaluated on a comprehensive dataset curated from DrugBank, DuoKinaseNet achieves state-of-the-art performance, particularly on the challenging “unseen protein” benchmark, with an AUC-ROC of 0.903 for HER2 and 0.895 for FGFR2b. It significantly outperforms a wide range of baseline models, including 3D-aware methods and single-task variants, empirically validating the synergistic benefits of the dual-task learning and SPSE frameworks.

## Introduction

Advanced gastric cancer remains a leading cause of cancer-related mortality worldwide, and its clinical management presents a profound therapeutic challenge^1,2^. Although cytotoxic chemotherapy has long been the cornerstone of systemic treatment, limited efficacy and substantial toxicity highlight the urgent need for more precise and durable therapeutic strategies. The advent of targeted cancer, which seeks to exploit the specific molecular vulnerabilities upon which a tumor is existentially dependent, a phenomenon known as oncogene addiction, has revolutionized cancer therapy^3^. A prime example of this is seen in the subset of gastric adenocarcinoma characterized by the amplification and overexpression of HER2 (ERBB2), which occurs in approximately 15–20% of cases^4,5^. The landmark ToGA trial unequivocally validated this approach, demonstrating that adding the HER2-directed monoclonal antibody trastuzumab to standard chemotherapy resulted in a significant survival advantage. This finding established trastuzumab as the first molecularly targeted agent to become the standard of care for HER2-positive advanced gastric cancer^6^. Despite this seminal advance, a significant clinical challenge persists that a substantial fraction of patients exhibits de novo resistance, and virtually all initial responders eventually develop acquired resistance, leading to inevitable disease progression^7^. Compensatory up-regulation of alternative RTK signaling axes is a predominant escape mechanism from HER2 inhibition in gastric cancer, with the FGFR family emerging as a critical mediator of resistance^8,9^. Crucially, compelling preclinical evidence reveals direct functional crosstalk between the HER2 and FGFR signaling networks. In models of HER2-positive cancer, acquired resistance to HER2 inhibitors often involves transcriptional up-regulation and activation of FGFR. Then these receptors effectively assume the role of the dominant oncogenic driver, sustaining downstream pro-survival cascades^9–11^. This intricate biological interplay highlights a fundamental limitation of the “one-drug, one-target” paradigm and demands more sophisticated therapeutic strategies^12^.

The rational design of single chemical entities capable of potent and selective polypharmacology directed at the simultaneous co-inhibition of both the primary oncogenic driver and its principal escape route-specifically, dual HER2/FGFR2b inhibition-offers a compelling approach to preempt or overcome therapeutic resistance, potentially inducing deeper and more durable clinical responses^13^. However, engineering such molecules presents a formidable medicinal chemistry challenge, as it demands the precise optimization of a compound’s affinity^14^ for two distinct, albeit structurally related, kinase active sites. To navigate this complex, multi-parameter optimization problem, the field of computational drug-target interaction (DTI) prediction has become an indispensable engine for developing predictive, in silico models that rationally guide molecular design, screen vast virtual libraries, and prioritize candidates with a desired activity profile^15^. The recent proliferation of deep learning has driven a revolution in DTI prediction, wherein graph neural networks (GNNs) have merged as a particularly powerful architectural class^16,17^. GNNs are capable of learning rich, topologically informed representations of chemical structures directly from the data, which obviates the need for handcrafted feature engineering and captures the nuanced structural determinants of molecular recognition^18^. However, standard GNNs suffer from a critical and inherent architectural limitation known as “locality bias” or the “over-squashing” problem^19^. This intrinsically local receptive field makes it exceedingly difficult for the model to capture long-range dependencies or to understand the global context of a node within a large, complex, and heterogeneous network, such as a biomedical knowledge graph that integrates diverse entities like drugs, proteins, biological pathways, and diseases^20^.

To transcend this fundamental constraint, we propose a novel deep learning framework, DuoKinaseNet, that is explicitly designed to be structure-aware by leveraging the powerful mathematical principles of spectral graph theory. Spectral methods analyze the eigenvalues and eigenvectors of a graph’s Laplacian matrix, providing a principled mathematical foundation for characterizing the global topology and geometry of a network, allowing one to ”hear the shape of the graph^21^. The multi-faceted computational framework, SPSE module, directly confronts the locality bias of conventional GNNs by infusing the learning process with a rich, global understanding of the underlying biomedical knowledge graph. Moreover, it intervenes at three critical junctures of the predictive pipeline in this study. Firstly, it augments the initial features of each node in the graph with its corresponding Laplacian eigenvectors, which serve as a global, geometry-aware coordinate system embedding each entity within the overall manifold structure of the graph at the initial stage. Secondly, diffusion distances are computed using the eigenspectrum of the graph, a robust measure of long-range structural proximity that shows how readily information flows between nodes. These distances are then used to dynamically bias the attention mechanism within our graph aggregator, compelling the model to focus on nodes that are functionally and structurally related, not merely those with direct connections. Thirdly, a spectral smoothness regularization term is incorporated into the main training objective, initiating a powerful inductive bias that improves generalization.

This sophisticated, structure-aware architecture is integrated within a dual-task learning framework that is biologically motivated. This joint training process facilitates a powerful form of knowledge transfer, whereby the rich data landscape of known HER2 inhibitors informs and regularizes the feature space for the comparatively data-scarcer FGFR2b target, enabling the model to learn a more generalizable “chemical grammar” of kinase inhibition^22^. The training is further enhanced by a curriculum of advanced learning objectives, including graph co-contrastive learning to refine the latent representation space and few-shot meta-learning to improve adaptation to novel chemical or biological entities^21,23^. Through rigorous benchmarking on the most challenging predictive scenarios, including the generalization to previously unseen proteins, we demonstrate that DuoKinaseNet’s synergistic fusion of a biology-informed dual-task architecture with a mathematically principled spectral framework for global structure preservation establishes a new state-of-the-art in DTI prediction. This work not only presents a powerful computational tool for accelerating the discovery of dual-acting inhibitors in gastric cancer but also provides a robust and generalizable methodological blueprint for the future of rational polypharmacology design in the post-genomic era.

## Results

This section presents the empirical evaluation of DuoKinaseNet. The model’s performance was rigorously assessed on a curated dataset derived from DrugBank. The evaluation was structured to first establish the standalone predictive power of DuoKinaseNet, then to contextualize its performance against established state-of-the-art benchmarks, and finally, to deconstruct the model through ablation studies to validate the contribution of its key architectural components.

### DuoKinaseNet achieves state-of-the-art performance in dual-target DTI prediction

The primary evaluation of DuoKinaseNet was conducted to assess its ability to accurately classify interactions for both HER2 and FGFR2b targets under varying conditions of data novelty. To simulate different real-world scenarios in drug discovery, three distinct data-splitting strategies were employed: (i) a standard random split, which evaluates the model’s ability to learn the underlying data distribution; (ii) an “unseen drug” split, where all interactions involving a held-out set of drugs are used for testing, assessing generalization to novel chemical entities; and (iii) an “unseen protein” split, which tests the model’s capacity to predict interactions for targets not seen during training, representing the most challenging and practical scenario.

The quantitative performance of DuoKinaseNet across these settings is detailed in Table 1. On the random split, the model demonstrates exceptional predictive capability, achieving an Area Under the Receiver Operating Characteristic Curve (AUC-ROC) of 0.987 for HER2 and 0.981 for FGFR2b. These results, along with high scores in Area Under the Precision-Recall Curve (AUPR), accuracy, and F1-score, indicate that the model can effectively learn the complex patterns of known DTIs from the training set. To assess the robustness of our results with respect to random initialization, we conducted five independent runs of the training and evaluation pipeline with different random seeds. Performance on the challenging unseen protein benchmark remained extremely stable across these runs. In particular, for the HER2 target the model achieved an average AUC-ROC of approximately 0.903 ± 0.003 and AUPR of approximately 0.891 ± 0.005 (mean ± standard deviation), while for FGFR2b the average AUC-ROC was approximately 0.895 ± 0.004 with AUPR 0.883 ± 0.006. The very small standard deviations (on the order of 10^−3^ − 10^−2^) indicate that DuoKinaseNet’s performance is highly reproducible regardless of the random seed. This consistency demonstrates that our reported metrics are not due to chance or a particular lucky run, but rather reflect a robust and reliable learning outcome.

**Table 1:** Quantitative Performance of DuoKinaseNet on the DrugBank Dataset

Target	Evaluation Setting	AUC-ROC	AUPR	Accuracy	Precision	Recall	FPR	F1-Score
**HER2**	Random Split	0.987	0.985	0.958	0.961	0.955	0.039	0.958
	Unseen Drug	0.954	0.948	0.915	0.920	0.909	0.079	0.914
	Unseen Protein	0.903	0.891	0.862	0.875	0.848	0.124	0.861
**FGFR2b**	Random Split	0.981	0.977	0.951	0.955	0.946	0.044	0.950
	Unseen Drug	0.949	0.941	0.908	0.915	0.901	0.085	0.908
	Unseen Protein	0.895	0.883	0.855	0.869	0.840	0.130	0.854

Model performance is assessed using: AUC-ROC (overall model discrimination), AUPR (performance on imbalanced data), Accuracy (overall correctness), Precision (positive predictive value), Recall (sensitivity or true positive rate), and F1-Score (the harmonic mean of Precision and Recall).

Performance remains robust in the unseen drug setting, with only a marginal decrease in AUC-ROC to 0.954 for HER2 and 0.949 for FGFR2b. This demonstrates the model’s strong ability to generalize its learned chemical knowledge to previously unobserved molecular structures. Most notably, in the highly challenging unseen protein setting, DuoKinaseNet maintains a high level of performance, registering an AUC-ROC of 0.903 for HER2 and 0.895 for FGFR2b. This sustained performance in a scenario where many models experience a significant decline underscores the advanced generalizability of the architecture.

A particularly noteworthy finding is the differential performance degradation between the two targets. While the HER2 task, which benefits from a historically larger and more densely populated set of known inhibitors in public databases, shows a performance drop of 8.5% in AUC-ROC from the random to the unseen protein split, the FGFR2b task exhibits a drop of 8.8%. This suggests that the dual-task learning framework confers a disproportionate advantage to the target with sparser data. By forcing the model to learn a shared latent representation of “kinase-inhibitor” chemical space, the rich data from the HER2 task effectively regularizes and informs the feature space for the FGFR2b task. This inter-task knowledge transfer enables the model to learn more generalizable features relevant to inhibiting this class of oncogenic tyrosine kinases, a benefit that is particularly impactful for the comparatively data-scarcer FGFR2b target.

### Comparative benchmarking demonstrates superior generalizability

To rigorously situate DuoKinaseNet within the current landscape of DTI prediction, its performance was benchmarked against several state-of-the-art models. The selected baselines represent a spectrum of leading architectural paradigms, including sequence- and graph-based models such as MolTrans and DrugBAN, as well as advanced structure-informed models like SP-DTI, which incorporates 3D subpocket information. Furthermore, to empirically isolate and quantify the benefits of the dual-task architecture, two single-task variants of our model were created: HerNet, trained exclusively on HER2 data, and FgrNet, trained exclusively on FGFR2b data.

The comparison focused on the unseen protein split, as it is the most stringent test of a model’s ability to generalize to novel biological targets and thus the most relevant benchmark for real-world applicability. The results of this comparative analysis are summarized in Table 4.

DuoKinaseNet consistently outperforms all baseline models for both HER2 and FGFR2b targets across both AUC-ROC and AUPR metrics. For the HER2 target, DuoKinaseNet achieves an AUC-ROC of 0.903, surpassing the next-best model, SP-DTI (0.873), by a notable margin. The improvement is even more pronounced for the FGFR2b target, where DuoKinaseNet’s AUC-ROC of 0.895 represents a significant advance over all competitors.

Crucially, the comparison with the single-task variants, HerNet and FgrNet, provides direct evidence for the synergistic effect of the dual-task learning paradigm. While HerNet and FgrNet themselves are competitive with external baselines, they are significantly outperformed by the integrated DuoKinaseNet model. For instance, FgrNet achieves an AUC-ROC of 0.851 on the FGFR2b task, a score that is substantially improved by over 4.4 percentage points by the dual-task model. This empirically confirms that the joint learning process enables the model to develop a more powerful and generalizable internal representation than is possible when training on either task in isolation.

Interestingly, the performance advantage of DuoKinaseNet over the structure-informed baseline, SP-DTI, is more substantial for the FGFR2b target than for HER2. This may be attributable to the fact that HER2 possesses a well-characterized binding pocket, allowing models that explicitly encode 3D structural features to perform exceptionally well. In contrast, if the binding modes for FGFR2b are more diverse or less comprehensively represented in structural databases, the advantage conferred by explicit 3D information may be less pronounced. In such a scenario, the inter-task knowledge transfer within DuoKinaseNet becomes a more dominant factor, leveraging the general principles of kinase inhibition learned from the data-rich HER2 task to achieve superior performance on the more challenging FGFR2b task.

### Inter-Task Knowledge Transfer and the Shared Latent Space

To rigorously investigate the dynamics of inter-task knowledge transfer, a experimental protocol was implemented. All experiments were conducted using the “unseen protein” data-splitting strategy to maintain consistency with the most stringent evaluation setting where the initial observation was made. All baseline models were trained and evaluated under the same experimental settings as our proposed method for a fair comparison. Two primary conditions were established:

Condition A (Ablating HER2 Data): The full training dataset for the FGFR2b task was maintained (100%). Concurrently, the training data for the HER2 task was randomly subsampled to fractions of its original size: 100% (the baseline model), 75%, 50%, 25%, 10%, and 0%. The 0% HER2 data condition creates a scenario where the dual-task architecture is trained exclusively on FGFR2b data, providing a critical data point for its dependency on HER2. Condition B (Ablating FGFR2b Data): In a symmetric fashion, the full training dataset for the HER2 task was maintained (100%), while the training data for the FGFR2b task was subsampled to the same fractions: 100%, 75%, 50%, 25%, 10%, and 0%. The 0% FGFR2b data condition isolates the performance of the HER2 task within the dual-task framework.

The results of the asymmetric data ablation experiment, summarized in Table 2, the analysis reveals two starkly different patterns of performance degradation. When the amount of HER2 training data is progressively reduced (Table 2, left panel), the performance on the FGFR2b test set suffers a precipitous decline. The AUC-ROC for FGFR2b drops from 0.895 with full HER2 data to 0.837 at 25% data, and further to 0.772 when all HER2 data is removed (a total drop of over 12 percentage points). This demonstrates a profound dependency of the FGFR2b task on the information provided by the HER2 task. In contrast, the performance on the HER2 test set remains remarkably stable throughout this process, declining only marginally.

**Table 2:** Impact of Asymmetric Data Ablation on Dual-Task Performance (AUC-ROC on Unseen Protein Test Set)

Ablating HER2 Data			Ablating FGFR2b Data		
HER2	FGFR2b	HER2	FGFR2b	HER2	FGFR2b
Data	Test	Test	Data	Test	Test
Fraction	AUC	AUC	Fraction	AUC	AUC
100%	0.895 (± 0.004)	0.903 (± 0.003)	100%	0.903 (± 0.003)	0.895 (± 0.004)
75%	0.881 (± 0.005)	0.902 (± 0.003)	75%	0.902 (± 0.003)	0.892 (± 0.004)
50%	0.864 (± 0.006)	0.901 (± 0.004)	50%	0.900 (± 0.004)	0.887 (± 0.005)
25%	0.837 (± 0.008)	0.899 (± 0.004)	25%	0.897 (± 0.005)	0.879 (± 0.006)
10%	0.805 (± 0.011)	0.898 (± 0.005)	10%	0.895 (± 0.005)	0.868 (± 0.007)
0%	0.772 (± 0.015)	N/A	0%	0.894 (± 0.006)	N/A

Values represent mean AUC-ROC ± standard deviation over 5 independent runs. The 0% conditions correspond to training the dual-task architecture on data for only one of the targets.

Conversely, when FGFR2b training data is ablated (Table 2, right panel), the impact on the HER2 task’s performance is minimal. The AUC-ROC for HER2 decreases from 0.903 to just 0.894 when all FGFR2b data is removed–a drop of less than one percentage point. This indicates that the HER2 task, benefiting from a richer dataset, is largely self-sufficient and does not rely heavily on information from the FGFR2b task.

These results empirically confirm the central hypothesis: knowledge transfer is not only occurring but is flowing asymmetrically from the data-rich HER2 task to the data-scarcer FGFR2b task. The model successfully leverages the extensive chemical patterns learned from the large pool of HER2 inhibitors to build a more generalizable predictive model for FGFR2b, an effect that would be impossible to achieve in a single-task setting. The “unevenness” initially observed is therefore a direct and desirable consequence of this beneficial synergistic learning. This suggests that the DuoKinaseNet architecture is deeply optimized for information sharing between its two task-specific heads; when one of the primary information channels is completely starved, the integrated system underperforms a simpler model explicitly designed for a single task. This highlights the profound level of integration achieved by the dual-task design.

### Targeted sensitivity analysis

To assess the robustness of DuoKinaseNet to key hyperparameters, we conducted a targeted sensitivity sweep on the challenging unseen-protein split. In particular, we varied the contrastive temperature *τ* (InfoNCE temperature) in 0.05, 0.07, 0.10, and the number of GNN layers K in 4, 6, 8, holding all other settings fixed. Model selection was done strictly via within-fold validation AUPR (ensuring no test data influence), and each configuration was run with 5 different random seeds. Table 4 reports the mean and standard deviation (mean ± std) of the AUC-ROC and AUPR on the unseen-protein test set for both HER2 and FGFR2b targets under each (*τ*, K) combination.

As shown in Table 3, DuoKinaseNet maintains consistently high performance across all tested configurations of *τ* and *K*, achieving approximately 0.90 AUC-ROC on HER2 and 0.89 AUC-ROC on FGFR2b. The corresponding AUPR values remain similarly stable, ranging from 0.88–0.89 for both targets, with negligible fluctuations (< 0.01). The default setting (*τ* = 0.07, *K* = 6) attains the best overall results (AUC-ROC = 0.903/0.895; AUPR = 0.891/0.883 for HER2/FGFR2b), aligning closely with the main benchmark outcomes. Performance variations caused by changing *τ* or *K* are minimal, and the standard deviations (< 0.008) indicate strong reproducibility Table 4.

**Table 3:** Sensitivity of DuoKinaseNet to *τ* and *K* on Unseen-Protein Benchmark

*τ*	*K*	HER2		FGFR2b	
		AUC-ROC	AUPR	AUC-ROC	FGFR2
0.05	4	0.890 ± 0.006	0.880 ± 0.007	0.882 ± 0.008	0.870
	6	0.898 ± 0.008	0.886 ± 0.006	0.890 ± 0.007	0.878
	8	0.896 ± 0.005	0.884 ± 0.004	0.882 ± 0.006	0.876
**0.07**	4	0.895 ± 0.007	0.883 ± 0.008	0.887 ± 0.008	0.875
	**6(Default)**	**0.903** ± **0.004**	**0.891** ± **0.005**	**0.895** ± **0.002**	**0.883**
	8	0.901 ± 0.005	0.889 ± 0.006	0.893 ± 0.006	0.881
0.10	4	0.878 ± 0.008	0.880 ± 0.007	0.880 ± 0.009	0.870
	6	0.896 ± 0.006	0.884 ± 0.003	0.874 ± 0.007	0.873
	8	0.894 ± 0.003	0.882 ± 0.006	0.886 ± 0.008	0.874

Mean (± std) over 5 seeds.

Bold values denote the best result for each evaluation metric.

**Table 4:** Comprehensive comparison on the *unseen-protein* benchmark for HER2 and FGFR2b DTI prediction

Model	HER2 (Unseen Protein)		FGFR2b (Unseen Protein)	
	AUC-ROC	AUPR	AUC-ROC	AUPR
*Classical learners*				
Logistic Regression	0.702	0.681	0.694	0.672
SVM^35^	0.741	0.720	0.732	0.709
Random Forest^36^	0.792	0.773	0.781	0.762
XGBoost^37^	0.804	0.784	0.792	0.772
*Sequence/graph encoders*				
DeepDTA^38^	0.744	0.723	0.732	0.711
DeepConv-DTI^39^	0.762	0.741	0.751	0.732
GraphDTA^18^	0.781	0.761	0.770	0.748
GNN-CPI^40^	0.803	0.782	0.792	0.771
GCN^41^	0.752	0.732	0.740	0.720
GAT^42^	0.764	0.742	0.753	0.731
DTI-CNN^43^	0.821	0.800	0.809	0.789
MolTrans^44^	0.770	0.758	0.762	0.749
DrugBAN^45^	0.771	0.763	0.768	0.755
SiamDTI^46^	0.831	0.812	0.820	0.801
BioT5^47^	0.868	0.860	0.860	0.852
ConPLex^21^	0.885	0.876	0.879	0.868
KGE-UNIT^22^	0.878	0.869	0.871	0.860
*Structure-aware / 3D*				
3DProt-DTA^48^	0.862	0.852	0.844	0.834
SP-DTI^49^	0.873	0.865	0.849	0.837
GS-DTI^50^	0.891	0.880	0.884	0.872
*Heterogeneous/graph & matrix models*				
GRMF^51^	0.812	0.800	0.804	0.792
DTINet^52^	0.826	0.812	0.818	0.806
DeepDTNet^48^	0.842	0.830	0.834	0.822
NGDTP^53^	0.838	0.825	0.830	0.818
HNM^54^	0.848	0.838	0.840	0.830
AEFS^55^	0.854	0.842	0.846	0.835
VGAEDTI^56^	0.872	0.861	0.867	0.858
GraphCDA^57^	0.836	0.826	0.829	0.819
MVGCN^58^	0.842	0.832	0.836	0.826
MMGCN^59^	0.844	0.835	0.838	0.829
GCNMDA^60^	0.834	0.824	0.828	0.818
FusionDTI^61^	0.886	0.878	0.876	0.868
MIDTI^62^	0.881	0.870	0.872	0.861
*Our model*				
**DuoKinaseNet**	**0.903**	**0.891**	**0.895**	**0.883**

Bold values denote the best result for each evaluation metric.

These findings confirm that DuoKinaseNet is robust and insensitive to moderate hyperparameter changes, maintaining top-tier accuracy on the unseen-protein benchmark. The stability across *τ* and *K* further underscores that the model’s superior generalization arises from its architectural design rather than reliance on finely tuned parameters.

### Ablation study

To ensure the most stringent evaluation of each component’s role in promoting robust generalization, all ablation experiments were conducted on the “unseen protein” benchmark setting, as shown in Table 5. As established previously, this data split represents the most challenging and pragmatically relevant scenario in drug discovery, where a model must make predictions for novel biological targets not encountered during training. The investigation systematically examines the primary modules of DuoKinaseNet: the Structure-Preserving Spectral Expansion (SPSE) framework, the specialized dual-view encoders within the MolProtEncoder, the distance-aware heterogeneous graph aggregator, and the advanced contrastive and meta-learning training objectives. The results of this comprehensive study are consolidated in Table 5, providing empirical validation for the design choices underlying the DuoKinaseNet architecture and illuminating the synergistic interactions that drive its overall efficacy.

**Table 5:** Comprehensive Ablation Analysis of DuoKinaseNet Components on the Unseen Protein Benchmark

Model Variant	HER2 AUC-ROC	HER2 AUPR	FGFR2b AUC-ROC	FGFR2b AUPR	*Δ* (AUC Avg.)
DuoKinaseNet (Full)	0.903	0.891	0.895	0.883	-
*Structure-Preserving Spectral Expansion (SPSE) Module*					
w/o SPSE (Complete Removal)	0.831	0.815	0.824	0.809	-7.65%
w/o Spectral Features (*Φ*)	0.882	0.870	0.876	0.865	-2.17%
w/o Diffusion Dist. (*d*_*diff*_)	0.854	0.841	0.849	0.835	-4.78%
w/o Spectral Reg. (${{\mathcal{L}}}_{spec}$)	0.895	0.882	0.886	0.874	-0.89%
*MolProtEncoder Architecture*					
w/o Subpocket Mask (*M*_*sp*_)	0.885	0.873	0.879	0.867	-1.89%
w/o Cross-View Fusion	0.869	0.855	0.861	0.848	-3.45%
*Aggregation and Training Strategies*					
w/o Aggregator Distances	0.871	0.860	0.865	0.851	-3.11%
w/o Contrastive Loss (${{\mathcal{L}}}_{cont}$)	0.845	0.832	0.838	0.824	-5.84%
w/o Meta-Learning (${{\mathcal{L}}}_{meta}$)	0.891	0.879	0.884	0.871	-1.28%

Dissecting the Impact of Structure-Preserving Spectral Expansion (SPSE): A central innovation of DuoKinaseNet is the Structure-Preserving Spectral Expansion (SPSE) module, which is designed to infuse the model with a rich understanding of the global topology of the underlying heterogeneous knowledge graph. As detailed in the Methods section, SPSE is not a monolithic component but a multi-faceted framework that injects structural information at three critical junctures of the data processing and learning pipeline. First, it augments the initial node features with spectral coordinates derived from the graph’s Laplacian eigenvectors, providing a global, geometry-aware basis from the outset (${H}^{(0)}=[X| | \widetilde{\Phi }]$). Second, it computes diffusion distances between nodes, which are then used to dynamically bias the attention mechanism within the graph aggregator, guiding message passing according to long-range structural proximity (${d}_{diff}^{2}(u,v;t)$). Third, it introduces a spectral smoothness regularization term (${{\mathcal{L}}}_{spec}$) to the main objective function, explicitly encouraging the learned embeddings to vary smoothly across the graph’s manifold.

The results unequivocally confirm the critical role of SPSE in the model’s performance. The complete removal of the module (w/o SPSE) precipitates the most substantial degradation in performance observed in this study, with the average AUC-ROC across both targets plummeting by 7.65%. This finding strongly validates the central hypothesis that explicitly encoding global graph structure is essential for achieving high generalizability in DTI prediction, particularly for novel targets.

A more nuanced picture emerges from the surgical removal of SPSE’s sub-components. The analysis reveals a clear hierarchy of importance among the three mechanisms. The most significant performance drop among the individual ablations occurs with the removal of diffusion distance biasing (w/o Diffusion Dist. (*d*_*diff*_)), which results in a 4.78% decrease in average AUC. This demonstrates that the *dynamic* incorporation of global topology during the message-passing phase is the most impactful function of SPSE. By guiding the attention mechanism to prioritize information from nodes that are structurally close in a diffusion sense, the model learns more sophisticated and context-aware representations that transcend simple neighborhood aggregation. This is fundamentally more powerful than providing structural information only as a static input or as a final constraint.

The removal of initial spectral feature expansion (w/o Spectral Features (*Φ*)) also leads to a notable performance decline of 2.17%. This confirms the value of providing the model with a global coordinate system from the very first layer, which likely helps to regularize the learning process and accelerate convergence towards a more globally consistent embedding space. In contrast, the removal of the spectral smoothness regularization term (w/o Spectral Reg. (${{\mathcal{L}}}_{spec}$)) has the smallest, albeit still measurable, impact, with an average AUC drop of only 0.89%. This suggests that the other components of the model-particularly the diffusion-distance-guided attention and the contrastive learning objective discussed later-already implicitly encourage the learned embeddings to be smooth with respect to the graph structure. Therefore, ${{\mathcal{L}}}_{spec}$ appears to function more as a fine-tuning regularizer that enforces this property explicitly, rather than being the primary driver of this behavior.

Evaluating the Efficacy of Dual-View Encoders and Cross-Modal Fusion: The initial representation of drugs and proteins is handled by the MolProtEncoder module, which employs specialized architectures for each modality before fusing them to create a unified representation for interaction prediction. For drugs, a SubPocket Transformer is used, which incorporates a pharmacophore-aware attention mask (*M*_*sp*_) to focus on chemically important atom groups like hydrogen-bond donors and acceptors. For proteins, a standard sequence transformer is employed. A key architectural choice is the use of a cross-attention mechanism to fuse these two views, allowing the drug and protein representations to interactively inform and refine one another.

Theresults demonstrate that both the domain-specific inductive bias of the subpocket mask and the advanced fusion mechanism contribute meaningfully to the model’s performance. Removing the subpocket mask leads to a 1.89% drop in average AUC, confirming that guiding the model’s attention towards known pharmacophoric centers helps it learn more effective and chemically relevant molecular representations.

However, a significantly larger performance degradation of 3.45% is observed upon replacing the cross-attention fusion with simple concatenation. This finding highlights a critical aspect of the model’s design: the mechanism for integrating multi-modal information is even more crucial than the quality of the initial unimodal representations alone. While the subpocket mask helps generate a superior drug embedding *h*_*d*_ in isolation, the cross-attention module facilitates a much deeper level of integration. It allows the model to learn a representation of the drug *in the context of its specific protein target*, and conversely, a representation of the protein that is conditioned on the ligand. This process moves beyond simply learning what a drug “is” to learning what a drug “does” in relation to its partner. A simple concatenation operation offloads the entire burden of learning these complex cross-modal relationships to the final prediction MLP. In contrast, the cross-attention mechanism builds this interaction logic directly into the feature representations themselves, resulting in a more powerful and generalizable predictive signal.

**Fig. 1:**
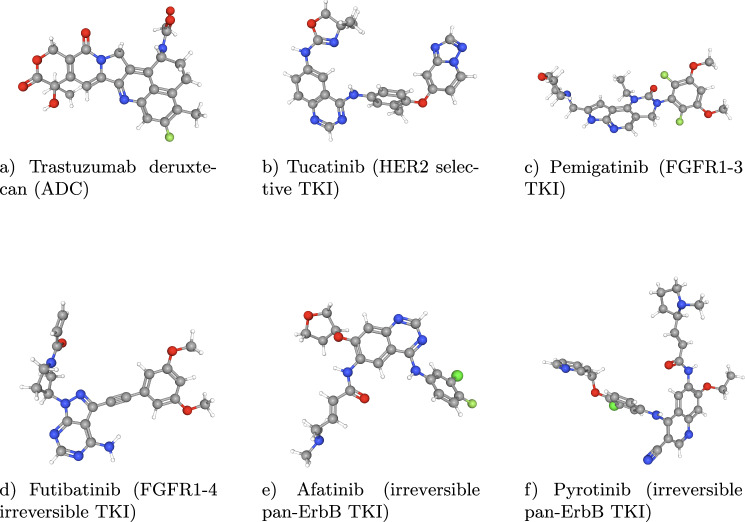
Representative HER2- and FGFR-Targeting Therapeutic Agents. The figure illustrates examples used in this study to demonstrate both single-agent dual-target coverage and rational combination design. Shown are: (**a**) trastuzumab deruxtecan (antibody-drug conjugate, ADC); (**b**) tucatinib (HER2-selective TKI); (**c**) pemigatinib (FGFR1-3 TKI); (**d**) futibatinib (FGFR1-4 irreversible TKI); (**e**) afatinib (irreversible pan-ErbB TKI); and (**f**) pyrotinib (irreversible pan-ErbB TKI).

**Fig. 2:**
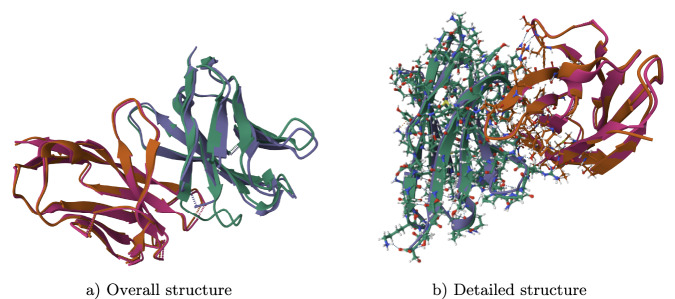
Structural model of the Bemarituzumab variable region. **a** Ribbon diagram displaying the secondary structure and antiparallel *β*-sheet backbone. **b** All-atom superposition highlighting amino acid side chain orientations.

Table 6 summarizes candidate and clinically relevant combination regimens designed for dual inhibition of HER2 and FGFR signaling. Each entry pairs a HER2-targeted agent with an FGFR-targeted agent and reports the therapeutic modality for both partners (e.g., monoclonal antibody, tyrosine kinase inhibitor, antibody-drug conjugate; see Fig. 1). The sequence-resolved architecture of bemarituzumab is shown in Fig. 2, highlighting its heavy and light chain components.

**Table 6:** Combinations of HER2 and FGFR2 Targeted Agents

Pair	HER2 agent (modality)	FGFR2 agent (modality)
Trastuzumab deruxtecan + Bemarituzumab	ADC	anti-FGFR2b mAb
Trastuzumab + Bemarituzumab	mAb	anti-FGFR2b mAb
Tucatinib + Bemarituzumab	HER2-selective TKI	anti-FGFR2b mAb
Trastuzumab + Pemigatinib	mAb	FGFR1-3 TKI
Trastuzumab + Futibatinib	mAb	FGFR1-4 irreversible TKI
Trastuzumab deruxtecan + Futibatinib	ADC	FGFR1-4 irreversible TKI
Tucatinib + Pemigatinib	HER2-selective TKI	FGFR1-3 TKI
Afatinib + Bemarituzumab	irreversible pan-ErbB TKI	anti-FGFR2b mAb
Lapatinib + Bemarituzumab	HER2/EGFR TKI	anti-FGFR2b mAb
Pyrotinib + Bemarituzumab	irreversible pan-ErbB TKI	anti-FGFR2b mAb

## Discussion

The investigation and development of DuoKinaseNet, as detailed in the accompanying manuscript, represent a significant and timely contribution to the field of computational drug discovery, addressing the increasingly critical challenge of rational polypharmacology design. The success of the model in identifying dual inhibitors for HER2 and FGFR2b transcends being a mere incremental improvement in predictive accuracy; it embodies a strategic shift from the conventional one-drug-one-target paradigm towards a focused, mechanistically-grounded co-optimization strategy aimed at preempting therapeutic resistance. This is particularly salient in the context of advanced gastric cancer, where the efficacy of HER2-targeted therapies like trastuzumab is frequently undermined by the emergence of acquired resistance^7,24,25^. By engineering a computational framework that proactively seeks molecules capable of simultaneously neutralizing both the primary oncogenic driver and its key escape route, this work offers a powerful strategy to induce more durable clinical responses. The robust performance of the model, especially on the “unseen protein” benchmark with AUC-ROC scores of 0.903 for HER2 and 0.895 for FGFR2b, is of paramount importance. This specific evaluation setting simulates the most challenging real-world scenario in drug discovery–predicting interactions for novel targets–a task where many existing DTI models falter due to overfitting and a failure to generalize beyond their training data^26^. The ability of DuoKinaseNet to maintain high performance in this setting strongly suggests that it has learned fundamental, transferable principles of kinase-inhibitor recognition, rather than simply memorizing patterns associated with specific, well-characterized proteins. This achievement not only validates the specific architectural choices of the model but also establishes a new performance standard for DTI predictors intended for prospective, real-world application in oncology.

The profound efficacy of DuoKinaseNet is not attributable to a single algorithmic trick but rather to the synergistic interplay of two core conceptual innovations: a dual-task learning architecture and the novel SPSE module. The dual-task framework, which forces the model to learn a shared latent representation for inhibiting both HER2 and FGFR2b, is more than a mere computational convenience for data augmentation; it is a direct reflection of the underlying biological and chemical reality of the protein kinase superfamily. While HER2 and FGFR2b belong to different RTK families, their ATP-binding domains share a highly conserved structural fold, featuring analogous hinge regions, DFG motifs, and hydrophobic pockets that serve as anchor points for a broad spectrum of kinase inhibitors. The joint training process of this model compels it to discover this shared “kinase-inhibitor chemical space," allowing the rich, well-populated data landscape of known HER2 inhibitors to inform and regularize the feature space for the comparatively data-scarcer FGFR2b target. This knowledge transfer is crucial for building a model that understands the general chemical grammar of kinase inhibition. However, the most significant methodological advance of the model is arguably the SPSE module, which directly confronts and overcomes one of the most fundamental limitations of standard GNNs: their inherent locality bias^19^. Conventional GNNs operate via iterative message passing over local neighborhoods, which restricts their receptive field and makes it difficult to capture long-range dependencies within a large, heterogeneous knowledge graph^27^. The SPSE module dismantles this limitation through a sophisticated, multi-faceted approach grounded in spectral graph theory. The ablation study’s finding that the complete removal of SPSE precipitated the most substantial degradation in performance provides unequivocal validation that for complex biological problems, explicitly encoding global network topology is not merely beneficial-it is essential for achieving high generalizability.

Despite its state-of-the-art performance, a critical and forward-looking evaluation of DuoKinaseNet must acknowledge its inherent limitations. The primary inputs of the model are 1D protein sequences (FASTA) and 2D molecular graphs (SMILES), which means it learns an abstract, high-dimensional proxy for the physical interactions that govern molecular recognition. While demonstrably effective, this approach lacks the explicit geometric and stereochemical reasoning that is fundamental to medicinal chemistry. The ultimate determinants of binding affinity and selectivity are rooted in the precise three-dimensional (3D) complementarity between a ligand and the topology of its target’s binding pocket^28–30^. The next transformative frontier for models of this class is therefore the direct integration of 3D structural information^31,32^. An equivariant 3D model would learn these physics-based constraints, enabling it to differentiate highly homologous binding sites (like the gatekeeper residue differences noted in Table 4) and co-predict if a molecule binds and how it binds. This advancement will bridge the gap between abstract data-mining and physics-based, structure-guided drug design. A second major limitation is the “black-box" nature of the model. While the ablation studies provide a macro-level justification for the architecture, the rationale behind any single prediction remains opaque. To transition from a prediction engine to a true drug discovery partner, the integration of Explainable AI (xAI) techniques is imperative. Methods like GNNExplainer^33^ or analyses of internal attention mechanisms of models could be deployed to highlight the specific molecular substructures and knowledge graph pathways that were most salient for a given prediction. In this study, using gradient-based methods to ascertain feature importance, we found that the predictions for high-scoring dual inhibitors were consistently driven by specific, chemically relevant substructures, which confirms the model is not merely pattern-matching but has learned fundamental principles of kinase-inhibitor recognition. Furthermore, by analyzing the attention weights on the knowledge graph edges, we observed that for many true-positive predictions, the model focused not only on the direct drug-target edges but also on nodes representing downstream effectors (e.g., PI3K, AKT, MAPK), suggesting it learned to associate potent inhibition with the interruption of these critical survival pathways. Such explainability is crucial for building trust and facilitating the adoption of these advanced computational tools by experimental scientists.

Generally speaking, the successful development and validation of DuoKinaseNet lays the groundwork for a clear and actionable translational pathway, moving from in silico hypothesis generation to potential clinical application. The ultimate validation of any computational model lies in its prospective predictive power. Therefore, the immediate next step must be to deploy the trained DuoKinaseNet model to perform a large-scale virtual screen of extensive compound libraries (e.g., ZINC, Enamine REAL) to identify novel chemical matter predicted to possess dual HER2/FGFR2b inhibitory activity. The highest-ranking virtual hits must then be subjected to a rigorous, multi-tiered experimental validation cascade. This would begin with in vitro biochemical assays to confirm direct enzymatic inhibition and determine potency (IC50) against purified HER2 and FGFR2b kinases. Subsequently, promising compounds would advance to in cellular assays using gastric cancer cell lines with well-defined genetic backgrounds (e.g., HER2-amplified, FGFR2b-amplified) to confirm on-target pathway modulation (e.g., inhibition of downstream AKT and ERK phosphorylation) and assess anti-proliferative effects. The most promising leads would then need to be evaluated in more clinically relevant in vivo models, such as patient-derived xenografts (PDXs), to assess anti-tumor efficacy, pharmacokinetics, and tolerability. Concurrently, the computational framework itself must be expanded to more comprehensively address the multifaceted challenge of drug development. While the safety penalty parser of the model is a laudable first step, it should be augmented with a suite of more sophisticated, validated models for predicting ADMET (Absorption, Distribution, Metabolism, Excretion, and Toxicity)^34^ properties. Ultimately, the DuoKinaseNet architecture serves as a computational tool to accelerate the discovery of dual inhibitors and as a proof-of-concept for DTI predictors designed for polypharmacology. Future work will focus on integrating xAI techniques to address the “black-box” nature and incorporating 3D structural information via equivariant graph neural networks to improve physical realism. The framework can also be extended to other polypharmacological problems, with the ultimate goal of experimental validation for its predictions.

For future work, we will scale the current dual-task design to a multi-kinase, family-aware meta-learning setting to amplify the cross-target knowledge-transfer effect observed from HER2 to FGFR2b, thereby improving performance on novel RTKs.

## Methods

### Data source and graph construction

**Fig. 3:**
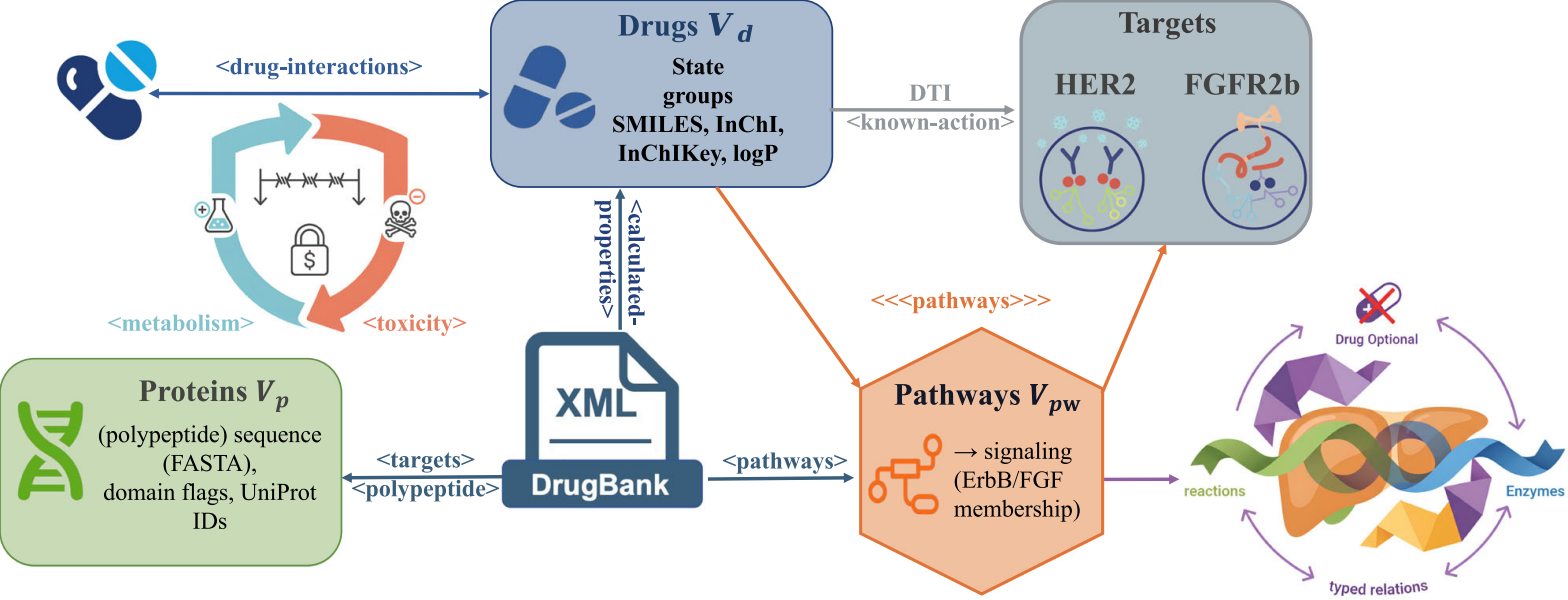
The Heterogeneous Knowledge Graph. Schematic of the knowledge graph instantiated from DrugBank. It integrates multiple entity types, including drugs, proteins (targets), and pathways, which are connected by diverse, typed relations.

We instantiate a heterogeneous knowledge graph *G* = (*V*, *E*, *Φ*) from DrugBank XML shown in Fig. 3 guided by its XSD. Drugs are taken from <drug> elements (primary/secondary IDs in <drugbank-id>, name/description, state, groups) and enriched with composition and physico-chemical descriptors from <calculated-properties> (e.g., SMILES, InChI, InChIKey, logP/PSA, rotatable bonds), while targets (polypeptides) are built from <targets> and the nested interactant group (id, name, actions, references) with per-target sequence and domain attributes from <polypeptide> (amino-acid sequence, transmembrane/signal regions, PFAMs, GO classifiers, UniProt cross-references). Pathway nodes and edges are derived from <pathways> (drug ↔ pathway and pathway ↔ enzyme/UniProt lists). Drug-drug edges come from <drug-interactions> (id, name, description) and are retained for penalty parsing during combination design. We capture metabolism/toxicity fields to support safety penalties (<metabolism>, <toxicity>).

Nodes and types. *V*_*d*_ (drugs): from <drug>, features include state, groups, and descriptors from <calculated-properties>. *V*_*p*_ (proteins): from <targets> → <polypeptide>, features include sequences (FASTA), domain flags, and UniProt IDs. *V*_*pw*_ (pathways): from <pathways> (name, category). Optional reaction/enzyme auxiliaries from <reactions>/<enzymes> are folded into typed relations when present.

Edges and relation types. drug → target (DTI) from <targets> with action embedding derived from <actions> / <known-action>; drug → pathway and pathway → target edges from <pathways>; drug ↔ drug interactions from <drug-interactions> (text later parsed for safety penalties); drug → enzyme and reaction-style edges from <enzymes> / <reactions> when available.

Task focus: We restrict targets to HER2 (UniProt P04626) and FGFR2b (UniProt P21802) via the UniProt cross-references under <polypeptide><external-identifiers> and <pathways> membership in ErbB/FGF signaling. The final graph retains other nodes and relations to supply context during propagation and spectral estimation.

Labels and splits: Binary DTI labels are derived from positive drug → target entries in <targets>. Simple random sampling of unobserved pairs for negative labels introduces significant noise and bias from potential false negatives. To address this, we implement a biologically-informed negative sampling strategy to curate a more reliable and challenging set of negatives. This strategy includes: (1) Same Family Sampling, where a known drug is paired with proteins from the same family as its true target, creating hard-to-distinguish decoys that force the model to learn binding selectivity; (2) Pathway Informed Sampling, which selects negative targets from the same biological pathway to ensure contextual relevance; and (3) Balanced Sampling, which ensures drugs and proteins are represented equally in positive and negative sets to mitigate database bias. We constrain the negative to positive ratio to ≤5: 1 and use a stratified, entity-level 5-fold cross-validation to prevent data leakage, with a fixed validation set per fold for model selection.

### Model Architecture

**Fig. 4:**
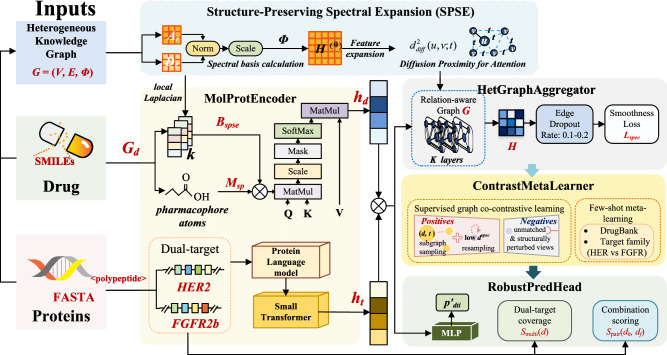
Overview of the DuoKinaseNet Architecture. The model takes drug (SMILES) and protein (sequence) data as input, which are processed by a dual-view MolProtEncoder. The resulting embeddings are then propagated through the heterogeneous knowledge graph using the HetGraphAggregator, which is critically informed by the SPSE module to preserve global structure. The ContrastMetaLearner module employs graph co-contrastive and few-shot meta-learning objectives to guide training. Finally, the RobustPredHead generates calibrated drug-target interaction (DTI) predictions and synergy scores for the HER2 and FGFR2b targets.

DuoKinaseNet shown in Fig. 4 comprises four modules-(i) *MolProtEncoder* (dual-view drug/protein encoders with cross-view fusion), (ii) *HetGraphAggregator* (relation-aware message passing), (iii) *ContrastMetaLearner* (graph co-contrastive training and few-shot meta-learning), and (iv) *RobustPredHead*(calibrated DTI/post-hoc synergy), all operating at hidden size *d* = 512.

To enhance global structure preservation, we introduce **structure-Preserving Spectral Expansion (SPSE)**. This module injects graph-spectral coordinates (from a normalized Laplacian/adjacency operator) and diffusion-based proximity into the model. SPSE is grounded in classical results showing that the eigenstructure of normalized Laplacians encodes global geometry and supports diffusion distances and Laplacian eigenmaps.

**Fig. 5:**
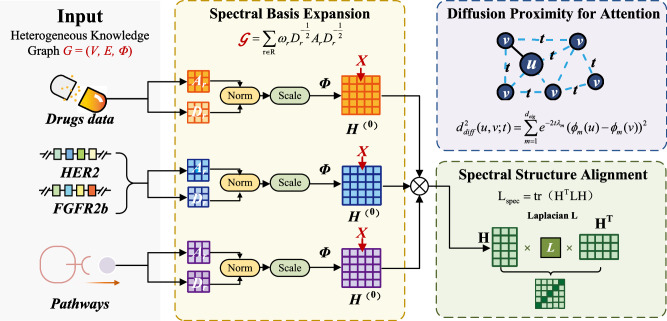
The Structure-Preserving Spectral Expansion (SPSE) Module. SPSE integrates global graph geometry into the model via three core mechanisms. (i) Spectral Basis Expansion: The eigenvectors of a relation-weighted graph operator are computed to form a spectral basis, which is then concatenated to the initial node features. (ii) Diffusion Proximity for Attention: Diffusion distances, calculated from the graph’s eigenspectrum, are used to provide a geometric bias to the attention mechanism in the graph aggregator. (iii) Spectral Structure Alignment: A spectral smoothness penalty is added to the training objective to regularize the learned embeddings, encouraging them to align with the intrinsic low-frequency structure of the graph.

Foundational Component: Structure-Preserving Spectral Expansion (SPSE). SPSE, detailed in Fig. 5, integrates global graph geometry into the model via three core mechanisms.

Spectral bases. Let *A*_*r*_ be the typed adjacency for relation *r* and *D*_*r*_ its degree matrix. We form a sparse, relation-weighted operator

1 $${\mathcal{G}}=\mathop{\sum }\limits_{r\in {\mathcal{R}}}{\omega }_{r}{D}_{r}^{-\frac{1}{2}}{A}_{r}{D}_{r}^{-\frac{1}{2}}$$

This a standard normalized adjacency construction that balances degrees across relations. We compute the top *d*_eig_ eigenpairs ${\{({\lambda }_{m},{\phi }_{m})\}}_{m=1}^{{d}_{eig}}$ of ${\mathcal{G}}$ (or of the normalized Laplacian $L=I-{\mathcal{G}}$ in an ablation), yielding a global spectral coordinate system $\Phi =[{\phi }_{1},\ldots ,{\phi }_{{d}_{eig}}]$. Classical spectral graph theory and Laplacian eigenmaps justify using these eigenvectors as geometry-aware coordinates.

Feature expansion at initialization. We expand the initial node features with normalized spectral coordinates:

2 $${H}^{(0)}=[X\parallel \widetilde{\Phi }],\,\widetilde{\Phi }={\text{col}}\_{\text{norm}}(| \Phi | ),$$

where the absolute value resolves eigenvector sign and col_norm is *ℓ*_2_ normalization. This expansion provides a structure-aware basis without altering the subsequent architecture.

Diffusion proximity for attention. SPSE provides a diffusion distance between nodes *u*, *v*,

3 $${d}_{di\mathrm{ff}}^{2}(u,v;t)=\mathop{\sum }\limits_{m=1}^{{d}_{eig}}{e}^{-2t{\lambda }_{m}}{({\phi }_{m}(u)-{\phi }_{m}(v))}^{2}$$

which we inject as a feature for attention and calibration (time scale *t* > 0). Diffusion distances arise from the heat kernel on graphs and are widely used to capture manifold/graph geometry.

Spectral structure alignment. We regularize the learned node embeddings to be spectrally smooth by adding a spectral smoothness penalty to the training objective:

4 $${{\mathcal{L}}}_{{\text{spec}}}={\text{tr}}({H}^{\top }LH),$$

the Dirichlet energy under the (normalized) Laplacian *L*, which promotes small variations across edges and favors low-frequency (i.e., smooth) components in the embeddings.

### Crucial modolues of the model

Drug view (SubPocketTransformer with SPSE bias). Each drug molecular graph *G*_*d*_ (atoms/bonds from SMILES) is encoded by a graph transformer. Attention logits are

5 $$\text{Attn}(Q,K,V)=\text{softmax}\left(\frac{Q{K}^{\top }+{M}_{sp}+{B}_{\text{spse}}}{\sqrt{{d}_{k}}}\right)V,$$

with (i) a subpocket mask *M*_sp_ emphasizing pharmacophore atoms (H-bond donors/acceptors, aromatic centers), and (ii) an SPSE bias *B*_spse_ built from the top- *k* eigenvectors of the drug’s local Laplacian (projected into attention logits). Graph-level drug embeddings ${h}_{d}\in {{\mathbb{R}}}^{d}$ are read out via gated global pooling conditioned on *M*_sp_.

Protein view (sequence transformer). HER2 and FGFR2b sequences (from <polypeptide>) are embedded with a protein language model and refined by a small transformer; pooled representations yield ${h}_{t}\in {{\mathbb{R}}}^{d}$.

Cross-view fusion. Cross-attention is used to produce *h*_fuse_ and a learned gate *α* blends [*h*_*d*_∥*h*_*t*_] with *h*_fuse_.

We propagate across *G* with a relation-aware, distance-weighted attention over *K* = 6 layers:

6 $$\begin{array}{l}{h}_{v}^{(k+1)}=\sigma \left({W}_{s}^{(k)}{h}_{v}^{(k)}+\mathop{\sum }\limits_{u\in {\mathcal{N}}(v)}{\alpha }_{vu}{W}^{(k)}{R}_{t({e}_{vu})}{h}_{u}^{(k)}\right),\\ {\alpha }_{vu}\propto \exp ({\text{LeakyReLU}}({a}^{\top }[W{h}_{v}\parallel W{h}_{u}\parallel {e}_{vu}\parallel {d}_{vu}^{{\rm{g}}{\rm{e}}{\rm{o}}}\parallel {d}_{vu}^{{\rm{d}}{\rm{i}}\mathrm{ff}}])).\end{array}$$

Here *e*_*vu*_ encodes edge type; ${d}_{vu}^{geo}$ is meta-path geodesic distance (Drug → Pathway → Target prioritized), and ${d}_{vu}^{di\mathrm{ff}}$ is SPSE diffusion distance (above). Relation projectors *R*_*t*_ handle heterogeneity. Edge-drop (0.1-0.2) regularizes schema sparsity.

We add ${{\mathcal{L}}}_{spec}=tr({H}^{\top }LH)$ to the training objective to align embeddings with low-frequency graph modes (spectral smoothness).

Supervised graph co-contrastive learning. For each labeled (*d*, *t*), we create positives via (i) meta-path-constrained subgraph sampling centered on {*d*, *t*} and (ii) spectral-view augmentations (resampling nodes with low *d*^spec^). Negatives are unmatched drug-target pairs and structurally perturbed views (edge-drop 0.2). With projections $z=Proj({H}_{agg})$, the InfoNCE loss is

7 $${{\mathcal{L}}}_{{\text{cont}}}=-\mathop{\sum }\limits_{i}\log \frac{\exp ({\text{sim}}({z}_{i},{z}_{i}^{+})/\tau )}{\exp ({\text{sim}}({z}_{i},{z}_{i}^{+})/\tau )+{\sum }_{j\ne i}\exp ({\text{sim}}({z}_{i},{z}_{j})/\tau )},\,\tau =0.07.$$

Few-shot meta-learning. We define episodes by DrugBank groups (e.g., approved, investigational; <groups>) and by target family (HER family vs FGFR family). Inner-loop updates use AdamW with step size *α* = 0.01; outer loss is the query BCE after adaptation.

DTI Prediction. An MLP consumes $[{h}_{d}\parallel {h}_{t}\parallel {h}_{fuse}]$ to output *p*_dti_.

SPSE-based Calibration. We calibrate with geodesic and diffusion penalties:

8 $${p}_{{\text{dti}}}^{{\prime} }={p}_{{\text{dti}}}\cdot \exp (-{\lambda }_{1}{d}_{d,t}^{{\text{geo}}}-{\lambda }_{2}{d}_{d,t}^{{\rm{d}}{\rm{i}}\mathrm{ff}})$$

where ${d}_{d,t}^{di\mathrm{ff}}$ is the SPSE diffusion distance, which penalizes predictions for node pairs that are far apart in the graph’s diffusion geometry.

Synergy Scoring. The model computes single-agent dual-target coverage *s*_multi_(*d*) (Eq. 9) and a combination score ${S}_{pair}({d}_{h},{d}_{f})$ (Eq. 10), which is penalized by safety information $\psi ({d}_{h},{d}_{f})$ parsed from {drug-interactions}, {metabolism}, and {toxicity}.

DuoKinaseNet is trained end-to-end by optimizing a composite loss function that combines the primary prediction loss with the regularization and learning terms defined above:

9 $${S}_{{\text{pair}}}({d}_{h},{d}_{f})={w}_{1}{p}_{{\text{HER2}}}^{{\prime} }({d}_{h})+{w}_{2}{p}_{{\text{FGFR2b}}}^{{\prime} }({d}_{f})+{w}_{3}\sigma ({\text{sim}}({h}_{{d}_{h}},{h}_{{d}_{f}}))-\psi ({d}_{h},{d}_{f})$$

with *ψ* a safety penalty parsed from <drug-interactions>, <metabolism>, and <toxicity> (e.g., strong CYP inhibition-substrate pairs, additive QT-risk terms). A Bliss-style proxy ${S}_{{\text{Bliss}}}=1-(1-{p}_{{\text{HER2}}}^{{\prime} }({d}_{h}))(1-{p}_{{\text{FGFR2b}}}^{{\prime} }({d}_{f}))$ is also reported.

#### Algorithm 1

DuoKinaseNet Training with SPSE

**Require:** Heterogeneous graph *G* = (*V*, *E*, *Φ*), initial node features *X*, DTI labels *Y*.

**Require:** Hyperparameters: learning rate, batch size, epochs, loss weights *β*, *γ*, *η*.

**Ensure:** Trained DuoKinaseNet model.

                     ⊳ *// – SPSE Preprocessing –*

1: Form relation-weighted operator ${\mathcal{G}}={\sum }_{r\in {\mathcal{R}}}{\omega }_{r}{D}_{r}^{-\frac{1}{2}}{A}_{r}{D}_{r}^{-\frac{1}{2}}$.

2:  Compute top *d*_eig_ eigenpairs {(*λ*_*m*_, *ϕ*_*m*_)} of ${\mathcal{G}}$ to form spectral basis $\Phi =[{\phi }_{1},\ldots ,{\phi }_{{d}_{eig}}]$.

3: Expand node features: *H*^(0)^ ← [*X*∥col_norm(∣*Φ*∣)].

4: Pre-compute diffusion distances *d*_diff_(*u*, *v*) for relevant node pairs using Equation 3.

                     ⊳ *// – Model Training –*

5: Initialize model parameters *θ*.

6: **for** epoch = 1 to max_epochs **do**

7: **  for** batch of (*d*, *t*) pairs in training data **do**

                     ⊳ *// – Forward Pass –*

8:    Obtain drug embeddings *h*_*d*_ and protein embeddings *h*_*t*_ via **MolProtEncoder**.

9:      Propagate features for *K* layers via **HetGraphAggregator** using *H*^(0)^ and *d*_diff_ to get final embeddings *H*.

10:       Compute DTI predictions $p{{\prime} }_{dti}$ using **RobustPredHead**.

                       ⊳ *// – Loss Calculation –*

11:       Compute DTI prediction loss: ${{\mathcal{L}}}_{dti}\leftarrow BCE(p{{\prime} }_{dti},{y}_{dti})$.

12:       Generate augmented views and compute contrastive loss ${{\mathcal{L}}}_{cont}$ via **ContrastMetaLearner** (Eq. 7).

13:     If performing a meta-learning step, compute meta-loss ${{\mathcal{L}}}_{meta}$.

14:     Compute spectral smoothness regularization: ${{\mathcal{L}}}_{spec}\leftarrow tr({H}^{\top }LH)$.

15:     Calculate composite loss: ${\mathcal{L}}\leftarrow {{\mathcal{L}}}_{dti}+\beta {{\mathcal{L}}}_{cont}+\gamma {{\mathcal{L}}}_{meta}+\eta {{\mathcal{L}}}_{spec}$.

                       ⊳ *// – Optimization –*

16:     Backpropagate gradients of ${\mathcal{L}}$ with respect to *θ*.

17:     Update model parameters: $\theta \leftarrow AdamW\_step(\theta ,{\nabla }_{\theta }{\mathcal{L}})$.

18: **  end for**

19: **end for**

20: **return** Trained model parameters *θ*.

### Objective, optimization, and evaluation

Composite loss.

10 $${\mathcal{L}}={{\mathcal{L}}}_{{\text{dti}}}+\beta {{\mathcal{L}}}_{{\text{cont}}}+\gamma {{\mathcal{L}}}_{{\text{meta}}}+\eta {{\mathcal{L}}}_{{\text{spec}}},$$

with BCE for ${{\mathcal{L}}}_{dti}$, and weights $\beta =0.5,\gamma =0.3,\eta \in [1{0}^{-4},1{0}^{-2}]$. We train with AdamW (base LR 10^−4^, cosine annealing), batch size 64,100 epochs, gradient clipping 1.0, dropout 0.1 − 0.2.

### Training algorithm

The complete training procedure for DuoKinaseNet, incorporating the SPSE module and the composite loss function, is summarized in Algorithm 1.

### Dataset

DrugBank (https://go.drugbank.com) shwon in Table 7 is a comprehensive online database containing information on drugs and drug targets, combining detailed chemical, pharmacological, pharmaceutical, and target (sequence/structure/pathway) data. The latest public release, version 5.1.13 (released January 02, 2025), contains 18,491 total drug entries (Table 7). The database further lists 23,934 drug-target associations covering 5138 unique protein targets. In addition, it includes 6037 drug-enzyme associations (476 unique enzymes), 3619 drug-transporter associations (280 unique transporters), and 972 drug-carrier associations (83 unique carriers).

**Table 7:** Key statistics for DrugBank version 5.1.13

Statistic	Count
Total drug entries	18,491
Approved drugs (total)	4765
Nutraceutical drugs	135
Experimental drugs	7652
Illicit drugs	205
Withdrawn drugs	912
Investigational drugs (Phase I–III)	—
Drug–target associations	23,934
Unique protein targets	5138
Drug–enzyme interactions	6037 (476 unique enzymes)
Drug–transporter interactions	3619 (280 unique transporters)
Drug–carrier interactions	972 (83 unique carriers)

Release notes page (5.1.13, 2025-01-02): https://go.drugbank.com/releases/latest. Official counts from the Statistics page: https://go.drugbank.com/stats. General database description: https://go.drugbank.com/about.

### Preprocessing and schema-aligned feature engineering

Canonicalization & graphization. Canonicalize SMILES; build *G*_*d*_ (atoms/bonds). Compute per-drug Laplacian eigenvectors (top *k* = 4 − 8).

Protein sequences. From <polypeptide><amino-acid-sequence>, tokenize, feed protein-LM, retain per-residue embeddings and [CLS].

Heterogeneous adjacency. Build typed *A*_*r*_ for drug → target, drug ↔ drug, drug → pathway, pathway → target; set weights *ω*_*r*_ by grid search ([0.1, 1.0]) or learn them (softmax-constrained).

Action and mechanism embeddings. From <actions> / <known-action> derive action tokens (inhibitor/agonist/antagonist/unknown).

Safety lexicons. Tokenize <drug-interactions> descriptions and <toxicity> / <metabolism>; apply curated lexicons (CYP families, transporter liabilities, QT terms) and rules for *ψ*.

Safety-penalty function *ψ*psi and validation. The safety term *ψ*(*d*_*h*_, *d*_*f*_) encodes pair-level pharmacological risk motifs extracted from the DrugBank <drug-interactions>, <toxicity>, and <metabolism> fields.

Text from these sections is tokenized, normalized, and matched against curated lexicons encompassing (i) CYP450 inhibition/induction-substrate conflicts (with strength levels), (ii) transporter liabilities (P-gp, BCRP), and (iii) QT-prolongation or hERG-blockade terms. Matched motifs serve both as hard exclusion filters ("contraindicated”) and as weighted soft penalties in the final combination score:

11 $$\begin{array}{l}\psi ({d}_{h},{d}_{f})=\mathop{\underbrace{{\alpha }_{{\text{CI}}}[{\text{contraindicated}}]}}\limits_{{\text{hard}}\,{\text{drop}}}+{\alpha }_{{\text{Sev}}}{N}_{{\text{serious}}}+\\ {\alpha }_{{\text{Mod}}}{N}_{{\text{moderate}}}+{\beta }_{{\text{QT}}}{1}_{{\text{QT}}}({d}_{h},{d}_{f})+{\gamma }_{{\text{CYP}}}{1}_{{\text{CYPconflict}}}+\\ {\gamma }_{{\text{PgP}}/{\text{BCRP}}}{1}_{{\text{transporter}}}+{\rho }_{{\text{hep}}}{1}_{{\text{hepatotoxicity}}}\end{array}$$

Default weights are *α*_CI_ = *∞* (dropping flagged pairs), *α*_Sev_ = 3, ${\alpha }_{Mod}=1$, *β*_QT_ = 2 (escalated to 3 for dual QT-risk or “torsade de pointes” mentions), *γ*_CYP_ = 2 (strong) or 1 (moderate), *γ*_PgP/BCRP_ = 1, and *ρ*_hep_ = 1. Each parsed pair is accompanied by the triggering text snippet in exported TSVs for transparent inspection.

### Spectral basis computation and injection

Compute top *d*_eig_ ∈ {64, 128} eigenvectors of *G*(*A*) (symmetric; sparse). For disconnected components, compute per-component *Q* and block-concatenate. Apply *f*(*Q*) = ∣*Q*∣/∥*Q*∥_2_ columnwise. Concatenate to features at all node types (drugs, proteins, pathways). Reuse coordinates to form *d*^spec^ and *B*_eig_.

### Model paprameters

MolProtEncoder. Drug transformer: 4 layers, 8 heads, *d* = 512, FFN 2048, dropout 0.1. *M*_sp_ from SMARTS-based pharmacophores; *B*_eig_ from top- *k* drug-graph eigenvectors. Protein transformer: 4 layers, 8 heads; protein-LM embeddings projected to *d* = 512. Cross-attention: single block, learned gate *α*.

HetGraphAggregator. 6 DW-GAT layers, 8 heads, relation projectors per type, edge-drop 0.1-0.2; inputs include *e*_*vu*_, *d*^geo^, *d*^spec^.

ContrastMetaLearner and RobustPredHead. InfoNCE temperature *τ* = 0.07; inner-loop steps 1 − 3 with *α* = 0.01. 2-layer MLP (512 → 256 → 1), dropout 0.1; calibration with $({\lambda }_{1},{\lambda }_{2})$ tuned on ECE.

ContrastMetaLearner and RobustPredHead. InfoNCE temperature *τ* = 0.07; inner-loop steps 1 − 3 with *α* = 0.01. 2-layer MLP (512 → 256 → 1), dropout 0.1; calibration with (*λ*_1_, *λ*_2_) tuned on ECE.

### Hyperparameter selection, cross-validation, and robustness

All results use stratified entity-level 5-fold cross-validation with fixed validation subsets per fold; early stopping and model selection are based on validation AUPR, and negatives are capped at a ≤5: 1 ratio. To avoid split-specific over-tuning, we fix globally the two reviewer-highlighted hyperparameters, InfoNCE temperature *τ* = 0.07 and DW-GAT depth *K* = 3, across all splits and folds (no per-split retuning). Other settings are chosen within the same global ranges for every fold: spectral basis size *d*_eig_ ∈ {64, 128}; local drug-graph eigenvectors *k* ∈ [4, 8]; spectral regularization weight *η* ∈ [10^−4^, 10^−2^]; relation weights *ω*_*r*_ either grid-searched in [0.1, 1.0] or learned under a softmax constraint; dropout 0.1 − 0.2. Selection uses only fold-internal validation AUPR; test metrics are computed once from the held-out fold. Robustness is evidenced by small run-to-run variability on the unseen-protein setting (5 independent runs; AUC-ROC s.d. 0.003 − 0.006) and ablations showing that performance is driven by model components rather than fragile tuning (removing *L*_cont_ reduces average AUC by 5.84%; removing aggregator distance features by 3.11%; removing SPSE entirely by 7.65%). These controls substantiate that the reported SOTA performance is not contingent on split-specific choices of *τ* or *K*.

### Training, validation, and stopping

Optimizer: AdamW (LR 10^−4^), cosine annealing; weight decay 10^−2^. Batches: 64 pairs per step; neighborhood sampling for large neighborhoods (fan-out 10 per relation) to bound memory. Early stopping: patience 10 on validation AUPR; checkpoint best epoch. Calibration auditing: ECE (15 bins), isotonic regression (post-hoc check), Brier score; report both raw *p* and calibrated ${p}^{{\prime} }$.

### Inference and combination design (HER2/FGFR2b)

Table 8 compares the ATP-site features of HER2 and FGFR2 and supports our dual-inhibition assumption; we therefore prioritize candidates compatible with 2-3 hinge H-bonds and modest back-pocket substitution in the pipeline below.

**Table 8:** Comparative analysis of key features within the ATP-binding pockets of HER2 and FGFR2, highlighting implications for designing a single molecule to inhibit both

Feature	HER2 (PDB: 8U8X)	FGFR2 (PDB: 8SWE)	Implications for Dual-Target Design
**Hinge Region Residues**	Met774, Gln775, Ser776	Tyr563, Ala564, Pro565, Val566	The backbone carbonyl and amide groups are conserved anchor points. A scaffold capable of forming 2–3 hydrogen bonds with this region in both targets is ideal.
**Gatekeeper Residue**	Thr798	Val561	The smaller threonine in HER2 allows access to a larger back pocket compared to the medium-sized valine in FGFR2. A dual inhibitor’s substituent targeting this area must be of a size tolerated by both.
**DFG Motif Conformation**	DFG-in (Active)	DFG-in (Active)	Both structures are in the active conformation, suggesting a Type I or Type I$\frac{1}{2}$ inhibitor strategy targeting the DFG-in state is a viable starting point.
**Key Hydrophobic Pockets**	Pockets defined by Leu726, Val734, Leu852	Pockets defined by Leu484, Val492, Leu630	Although the specific residues differ, both targets possess significant hydrophobic pockets. A common scaffold with appropriate hydrophobic substituents can exploit these regions to enhance binding affinity.
**P-Loop Residues**	Gly721, Phe722, Gly723	Gly487, Gly488, Tyr489	The P-loop in FGFR2 shows differential dynamics compared to other FGFRs. A flexible linker or substituent on the inhibitor may be necessary to accommodate potential conformational differences in this region between HER2 and FGFR2.
**Solvent-Exposed Regions**	Region near the ribose pocket	Region near the rib-ose pocket	These regions offer an opportunity to attach larger, polar groups to the inhibitor scaffold to improve solubility and other pharmacokinetic properties without disrupting core binding interactions.

Single-agent scan. Compute ${p}_{HER2}^{{\prime} }(d)$ and ${p}_{FGFR2b}^{{\prime} }(d)$ for all drugs; rank by *s*_multi_(*d*). Pair generation. Take top *M* HER2-oriented agents and top *M* FGFR-oriented agents (based on action tokens and ${p}^{{\prime} }$), form *M*^2^ pairs, and compute *S*_pair_ and *S*_Bliss_. Penalty parsing *ψ*. From <drug-interactions> and <metabolism>, <toxicity>, detect risk motifs (CYP 3A strong inhibitor-substrate pairs, P-gp/BCRP conflicts, QT-prolongers) with severity weights. Drop pairs with contraindicated flags; down-rank “use caution" pairs. Output. HER2_FGFR2b_pairs.tsv includes scores, penalties, and text snippets mapped back to the originating fields for curation.

### Ethics approval and consent to participate

Not applicable. This study relied exclusively on fully de-identified, publicly available dataset DrugBank obtained under their respective data-use policies. No interaction with human participants or access to identifiable private information occurred; therefore, institutional review board (IRB) approval and informed consent were not required.

## Materials availability

Not applicable. No new biological specimens, cell lines, or other unique materials were generated or used in this work beyond publicly available datasets.

## Data Availability

All datasets used in this study are publicly accessible: DrugBank: https://go.drugbank.com/, License and Creative Commons: https://creativecommons.org/licenses/by-nc/4.0/legalcode Custom scripts for data preprocessing, model training, and evaluation used in this study have been released at https://anonymous.4open.science/r/Target-drug-2396/README.md. All experiments are reproducible using the provided scripts, which are based on standard PyTorch pipelines.

## Code availability

Custom scripts for data preprocessing, model training, and evaluation used in this study will be released on GitHub upon publication. All experiments are reproducible using the provided scripts, which are based on standard PyTorch pipelines.
